# Displacement and Emotional Well-Being among Married and Unmarried Syrian Adolescent Girls in Lebanon: An Analysis of Narratives

**DOI:** 10.3390/ijerph17124543

**Published:** 2020-06-24

**Authors:** Sophie Roupetz, Susan A. Bartels, Saja Michael, Negin Najjarnejad, Kimberley Anderson, Colleen Davison

**Affiliations:** 1 Department of Medical Psychology and Medical Sociology, University of Leipzig, 04103 Leipzig, Germany; sophie.roupetz@gmail.com (S.R.); kimberley.anderson@medizin.uni-leipzig.de (K.A.); 2 Department of Emergency Medicine, Queen’s University, Kingston, ON K7L 4V7, Canada; susanabartels@gmail.com; 3 Department of Public Health Sciences, Queen’s University, Kingston, ON K7L 3N6, Canada; 4 ABAAD Resource Center for Gender Equality, Beirut, Lebanon; sajamichael@gmail.com (S.M.); negin.njn@gmail.com (N.N.); 5 Department of Social Sciences, York University, Toronto, ON M3J 1P3, Canada; 6 Department of Global Development Studies, Queen’s University, Kingston, ON K7L 3N6, Canada

**Keywords:** displacement, adolescent, emotional well-being, Syria, Lebanon, refugee, girls, emergency settings, migrant, health

## Abstract

Lebanon hosts over one million refugees displaced from Syria as a result of the armed conflict—of whom, approximately 15% are adolescents aged between 12 and 17 years of age. Many female adolescent migrants report a decrease in quality of life and an increase in family tensions. This study sought to investigate the emotional well-being of adolescent Syrian girls in Lebanon. We hypothesized that married girls may experience additional hardships and thus greater feelings of dissatisfaction in daily life, given their young marriage and responsibilities at home. This study was part of a large mixed-methods study on the experiences of Syrian refugee girls in Lebanon (*n* = 1422). Using line-by-line coding and thematic analysis, 188 first-person narratives from Syrian girls were analysed. Our results highlight poor emotional well-being among married and unmarried girls, with sadness, fear and anger commonly mentioned. Some participants expressed feelings of hope, happiness, gratefulness and empowerment. Unmarried girls (*n* = 111) were more likely to associate their shared stories with negative feelings such as sadness (47% vs. 22%), disappointment (30% vs. 19%), and frustration (32% vs. 22%) than were married girls (*n* = 77). Four themes emerged as important determinants: access to education, perceived safety, peer support, and longing for life back in Syria. Continued efforts to improve emotional well-being for married and unmarried refugee girls are needed in Lebanon, in particular those that address the nuances for these groups.

## 1. Introduction

### 1.1. The Syrian Conflict and the Experience of Syrian Refugees

Since the beginning of the Syrian conflict in 2011, war and forced migration have resulted in the exodus of over 5 million Syrian refugees to neighboring countries. Lebanon hosts the largest number of refugees per capita in the world, with approximately 1 million Syrian refugees currently registered—of whom, approximately 80% are women and children and almost 60% are school aged [1]. Lebanon was considered part of “Greater Syria” under the Ottoman Empire. In 1976, the Syrian occupation of Lebanon, which began during the Lebanese civil war and lasted for 29 years, strained relations between the two countries. It was not until 2008 that Syria officially recognized Lebanon’s sovereignty. The long history between Syria and Lebanon, as well as the experience of hosting Palestinian refugees since 1948 [2], influenced the Lebanese government’s response to the influx of Syrian refugees [3,4]. Many displaced Syrians were not and still are not currently able to register as refugees with the United Nations, and unregistered Syrians in particular report facing a myriad of obstacles in regard to accessing health services, employment, and education [5,6]. Some refugees live in informal tented settlements while others are more integrated into the host population, though often in substandard housing in urban neighborhoods [7]. Several studies highlight the exacerbated long-term negative effects of adverse living conditions on refugees [8,9,10]. Displacement often reduces access to effective service delivery, destroys national and personal assets, and undermines livelihoods [11]. The Syrian humanitarian crisis has altered every aspect of migrants’ daily life [12], with the resulting socio-economic and political instability creating an environment that renders children increasingly vulnerable. For children exposed to warfare and under a constant threat of violence, stressors can have a significant effect on emotional well-being and mental-health including anxiety, depression and post-traumatic stress disorder (PTSD) [13,14], especially for girls, who are vulnerable to sexual exploitation and other forms of violence. Rates of child marriage have increased, with the age of marriage decreased [15,16,17], and it is likely that marriage experiences affect the emotional well-being of young migrants, though this is less well explored. Emotional well-being refers to a person’s positive or negative affect and their general satisfaction with life [18]. The frequency and intensity of emotions such as joy, sadness, frustration or anger influence the pleasant or unpleasant nature of an individual’s life [19,20]. Insufficient attentiveness to emotional well-being among young female refugees can have long-term negative consequences for both the individual and society at large. However, there is a substantial gap in knowledge and understanding around the emotional well-being of forcibly displaced adolescents. 

This study aimed to generate a better understanding of emotional well-being among married and unmarried female adolescent Syrians displaced to Lebanon. Our objective was to explore a possible link between the migrant experience and girls’ emotional well-being, and whether there was a difference in the emotional well-being of married versus unmarried girls. While expecting that many refugee girls might report negative emotions related to daily challenges such as access to education and safety, we also hypothesized that married girls would express additional negative feelings related to their young marriage and increased responsibilities at home and with child rearing. Given the overall resilience of children, even in devastating situations, we also expected to see expressions of positivity.

### 1.2. The Experience of Adolescent Syrian Girls Displaced in Lebanon

Adolescence is a critical period in one’s development, with particular vulnerability among those who are displaced [21]. In total, an estimated 15% of Syrian refugees in Lebanon are between 12 and 17 years of age [1]. Young Syrians displaced to Lebanon report a decrease in their quality of life, increased tensions within the family and feelings of a lack of safety. Alarmingly, one study revealed that approximately 41% of young Syrians had thought about committing suicide since the beginning of the Syrian refugee crisis [12]. The exposure of adolescents to violence and war increases the risk of mental health problems [22], with PTSD and depression being most prevalent [23]. Due to often undesirable living conditions, young refugees are more likely to develop behavioral or emotional problems, including aggression and other affective disorders [24]. For these reasons, there is an overriding need for special attention to the emotional well-being of adolescents and their unique needs within the Syrian humanitarian response [7]. Forced migration often compromises the rights of children and adolescents while also creating an environment that impedes growth and does not allow children to positively thrive. In humanitarian crises, female adolescents are among the most vulnerable to these damaging circumstances. Displacement is commonly related to a high rate of dropping out of education; approximately 40% of female Syrian girls aged 15 to 18 years had been enrolled in formal education in their home country [25], but did not continue in Lebanon mainly due to cost of education and incompatibility of the school curriculum compared to the syllabus used in Syria [12]. In addition, a majority of displaced Syrians report having experienced physical or verbal violence in Lebanese schools [25]. Some Syrian girls are out of school due to marriage. In the Lebanese context, poor socio-economic conditions and fear for girl’s safety has led some families to marry their daughters off at a younger age [15]. Those girls marrying young, and particularly those with older spouses, can be at increased risk of intimate partner violence [26,27,28]. Restrictions to education and a lack of safety make them dependent on men, resulting in a lack of agency and the continued presence of patriarchal pressures associated with child marriage [7,15]. However, some female Syrian refugees have gained more decision-making power and responsibility within the family and community since the Syrian war began [29] as a result of men who have been killed or captured in combat, or men who have migrated outside of the country separately. These are new anomalies as, prior to the crisis, Syrian men dominated in almost all aspects of society [30]. We take a critical feminist perspective on our analysis of the crisis, and women’s well-being within it, and highlight past ideological and institutional structures that disadvantaged women and girls as well as possible systematic changes within the past decade [31]. An intersectional feminist approach looking at gender in circumstances of forced migration also frames our work. We understand gender beyond the dominant focus on women and maternity only. For instance, when women are centered in work on migration, they are often constructed as mothers, wives, daughters and not as community leaders, public persons or workers and defined primarily through their compulsory positioning in the hetero-patriarchal family [32,33]. Displaced Syrian refugee women and girls in Lebanon face precarity and are more likely exposed to gender-related problems such as harassment and exploitation than Lebanese or non-refugee women [34]. In Lebanon, some Syrians are only allowed to work in agriculture, construction and environment while others registered with the Office of the United Nations High Commissioner for Refugees (UNHCR) have no opportunity to work [35,36,37]. Local employers and recruiters are known to exploit the lack of socio-legal status to impose substandard working conditions on workers, particularly those working without permission. This can lead to the emergence of additional forms of gender-based violence (GBV), such as human trafficking of young girls and women for the sex trade. Therefore, labor exploitation of Syrian women and girls is to be understood within the wider framework of a lack of freedom of movement, precarious livelihoods and a precarious socio-legal status [38]. 

Parental support is crucial for a child’s adjustment to war stress and a fundamental protective moderator for child outcomes [39,40]. However, displaced Syrian families struggle with isolation from their original support groups [41] and parents face overwhelming emotional challenges. Parental stress and family violence play a key role in the emotional and behavioral outcomes of displaced Syrian children [42]. Studies found that some factors such as family cohesion, family support and parental psychological health; individual dispositional factors such as adaptability, temperament and positive esteem; and environmental factors such as peer and community support, temper or aggravate poor psychological health [43]. Tailored parenting programs for displaced families do have significant benefits for both the migrant children and their parents [42]. Additionally, tense relationships with Lebanese peers and concerns about harassment and physical violence by members of the host community have been reported by Syrian refugee girls [7]. Crime rates are reportedly high in refugee settlements as a result of a scarce police presence, further enabling racism and harassment to thrive, which can mean that refugees, and young girls in particular, fear for their safety [44]. Subsequent feelings of fear, anger, boredom, despondency, pessimism, loss of control and frustration severely affect the psychosocial health and emotional well-being of displaced adolescent girls [12]. The ultimate goal of this work was to more fully understand the experiences and emotional responses of displaced Syrian girls, both married and unmarried, to inform tailored strategies and interventions that will better support them and their families.

## 2. Materials and Methods

### 2.1. Study Design and Participants

This analysis is derived from a larger cross-sectional, mixed-methods study on the experiences of displaced Syrian girls in Lebanon conducted from July to August 2016 by researchers from Queen’s University, Canada, and the ABAAD Resource Center for Gender Equality, Lebanon. In total, 1422 self-interpreted narratives were collected by trained interviewers in three regions hosting the majority of displaced Syrians in Lebanon: Beirut, Beqaa and Tripoli. A variety of participant subgroups were intentionally included: married and unmarried Syrian girls; Syrian parents; Syrian, Lebanese and Palestinian men (both married and unmarried); and community leaders. Individuals had to be at least 13 years of age to be eligible to participate in the overall study. A convenience sample of potential participants was recruited in their naturalistic environments (cafes, shops, transportation depots, points of service delivery, etc.).

### 2.2. Data-Collection Tool

Data were collected using a mixed-methods, digital data-collection tool called SenseMaker (Cognitive Edge, Singapore). The tool has been shown to be efficient for collecting data in humanitarian settings [16,45]. It allows the collection of perspectives on sensitive topics [16]. Brief anecdotes from a large number of people can be collected in a relatively short time period while also providing contextualization of the quantitative data [46,47]. The narrative-based research methodology can be delivered using the SenseMaker app on a tablet offline. Participants who gave informed consent were asked to share a story, in response to one of three story prompts, about the experiences of Syrian girls in Lebanon. After audio recording the shared narrative in Arabic, participants ‘plotted’ their interpretation of the shared experience by responding to predefined questions on handheld tablets. Quantification of the locations of plotted points in space (on a line, in a triangle or in a rectangular grid) provided statistical data linked to the qualitative narrative [47]. A series of multiple-choice questions were also asked to collect demographic data and to help contextualize the shared narratives. One multiple-choice question that was key to the current analysis asked, “How does this story make you feel?” with six possible positive responses (encouraged, good, happy, hopeful, relieved, and satisfied) and six possible negative responses (angry, disappointed, embarrassed, frustrated, sad, and worried). Each participant could choose up to three emotions. More detailed information on the survey design and data-collection procedure can be found in Bakhache et al. [16], and data from the larger study can be found in Bartels et al. [15]. 

### 2.3. Ethical Considerations

The interviewers were trained on research methods, ethics, data collection and basic introduction to issues of gender-based violence (GBV). The interviewers were carefully selected to ensure that gender and nationality aligned with those of the intended participants. Information about the study and the study consent statement was read and reviewed by study participants in Arabic and then ascertained by tapping a consent box on the tablet. All interviews were conducted anonymously and confidentially without collecting any identifiers, and participants were asked to use aliases when recording their stories. Identifying material such as specific names or locations mentioned in the stories were omitted upon transcription and translation. No monetary incentives were offered since the survey was brief and participants were interviewed in their naturalistic environments. In cases of identified adverse events or GBV-related incidents, interviewers were trained to report issues to the relevant ABAAD field officer and project team member (three officers were assigned for each of three research implementation sites), who in turn would report and discuss issues with the project manager to decide on the best referral mechanism route. This protocol was established to ensure participant support in the case of any adverse situations; no events were reported during the study. The Queen’s University Health Sciences and Affiliated Teaching Hospitals Research Ethics Board approved the study protocol (#6020027).

### 2.4. Analysis

#### 2.4.1. Quantitative Data

SenseMaker data were exported to Tableau (Mountain View, California, CA, USA) for visual examination of overall response patterns, and to SPSS (SPSS Inc., Chicago, IL, USA) for statistical analysis. Descriptive analyses were conducted to characterise study participants and responses to survey questions. These were typically in the form of categorical variables (yes/no and multiple choice) and, thus, frequencies and proportions are reported. Questions that appeared to have potential differences based on visual inspection of the triads were selected for further analysis. For the triad questions, geometric means and their accompanying 95% confidence ellipses were calculated [using R (R Foundation for Statistical Computing, Vienna, Austria) statistical scripts] to identify differences in how married and unmarried girls responded [48]. The geometric mean, rather than the more familiar arithmetic mean, is the appropriate average to use for a group of story dots (data points) in a triangular diagram [49,50]. A 95% confidence ellipse provides a statistical estimate of the boundary inside which we expect the mean for a particular group or cohort to fall. Thus, two ellipses that do not overlap at all have a 95% chance of being statistically distinct [51]. 

#### 2.4.2. Qualitative Data

Audio-recorded narratives were professionally transcribed and translated from Arabic to English. We analysed all first-person narratives shared by Syrian girls aged 13–17 who had been in Lebanon for five years or less (*n* = 188). Inductive thematic analysis was conducted in four phases, as proposed by Braun and Clarke [52]. First, authors (S.R., N.N., C.D., S.M. and K.A.) familiarised themselves with the data by reading all 188 first-person stories multiple times. Second, coders identified all narratives that contained phrases or words expressing emotions or mentioning emotional well-being. The extracts were then coded line by line and given initial descriptive codes (e.g., regretting to be married early, happiness with friends, afraid at night). These codes helped to deconstruct the text into identifiable units of meaning and descriptive labeling placed on the raw data. These initial codes were first independently identified by coders (authors S.R., S.M.) and a subset of the stories was duplicate-coded (by authors N.N. and C.D.). An assessment of intercoder agreement was carried out, where researcher (C.D.) read through the coded narratives and discrepancies were discussed with the other coders. Third, these codes were conceptualised into interpretive analytical categories (situations of humiliation, fear, empowerment) to help identify and explain the relationship between the codes and identify areas of consensus and areas of divergence. Categories of codes helped explain the meaning and context of emotional feelings being expressed [53,54]. Categorization helped organize the initial codes into meaningful patterns [55]. An inductive approach was used to do this, to allow categories to emerge from the data in a bottom–up process. Final codes and categories were again decided by consensus as per Hill, Thompson and Williams [56]. In the fourth and final phase of the qualitative analysis, excerpts for each of the identified categories were culminated and reviewed.

#### 2.4.3. Quantitative and Qualitative Data Synthesis

The qualitative and quantitative findings were reviewed in parallel. The research team used analytic memos and discussion to note patterns across and within categories and data types. Four overarching themes emerged from this comprehensive review and these represent the synthesis of the quantitative and qualitative findings. 

## 3. Results

Table 1 outlines the socio-demographic characteristics of the sample; including age, marital status, location in Lebanon and time spent in Lebanon.

**Figure 1:**
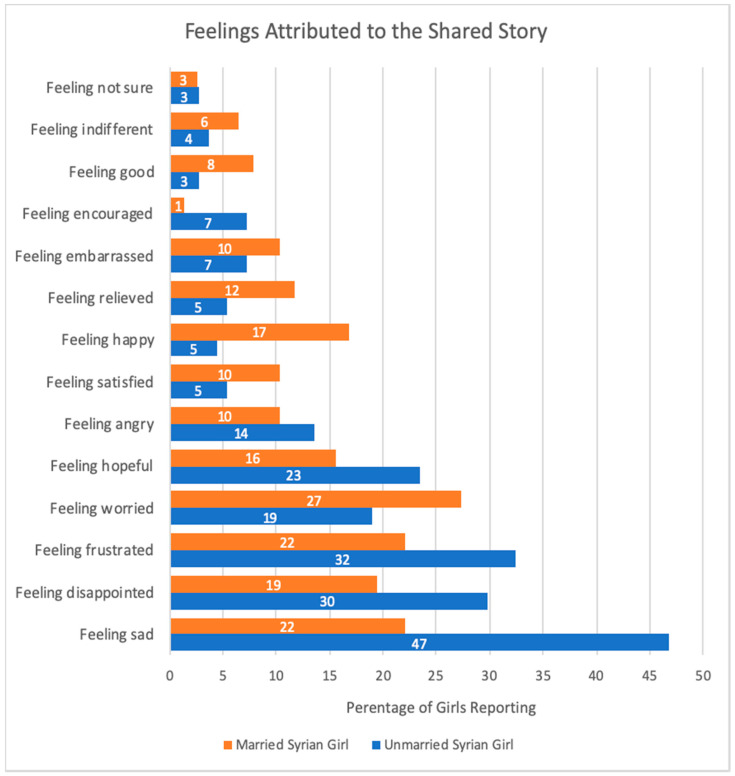
Proportion of married (*n* = 77) and unmarried (*n* = 111) girls who self-reported specific feelings attributed to the story they shared. The question was “how does the story make you feel?”

Findings from our study show that migrant Syrian girls have faced difficult living conditions in Lebanon and have been exposed to diverse challenges in school, at work and in their communities. Girls were exposed to physical, sexual, and racially motivated violence and many had concerns about their own safety. We collected information on emotional well-being in two ways from married (*n* = 77) and unmarried (*n* = 111) girls—first, by qualitatively coding the content in the girls’ stories, and second by asking the girls themselves how their story made them feel. As illustrated in Figure 1, for both married and unmarried girls, negative emotions such as sadness, frustration or anger were more often chosen in response to the question “how does the story make you feel?”. However, unmarried girls were more likely to associate their stories with negative feelings than were married girls. For example, unmarried girls were more likely to report that they felt sad (47% vs. 22%), disappointed (30% vs. 19%), and frustrated (32% vs. 22%) in comparison to married girls. In contrast, married girls were more likely to report that they felt worried (27% vs. 19%), happy (17% vs. 5%), and relieved (12% vs. 5%) in comparison to unmarried girls. 

A majority of the interviewees communicated negative emotions as assessed through the qualitative coding of their micro-narrative. For instance, frustration and anger were highly prominent, followed by feelings of fear, shame, sadness and loneliness. In contrast, some participants did communicate positive emotions and expressed feelings of hope, as well as happiness, gratefulness and empowerment. After coding for emotions and examining their context and determinants, four overarching themes emerged as important: access to education, perceived concerns about safety, peer support and longing for life back in Syria. Within these themes, differences in the emotional response of married and unmarried girls are noted.

### 3.1. Theme 1: Education

**Figure 2:**
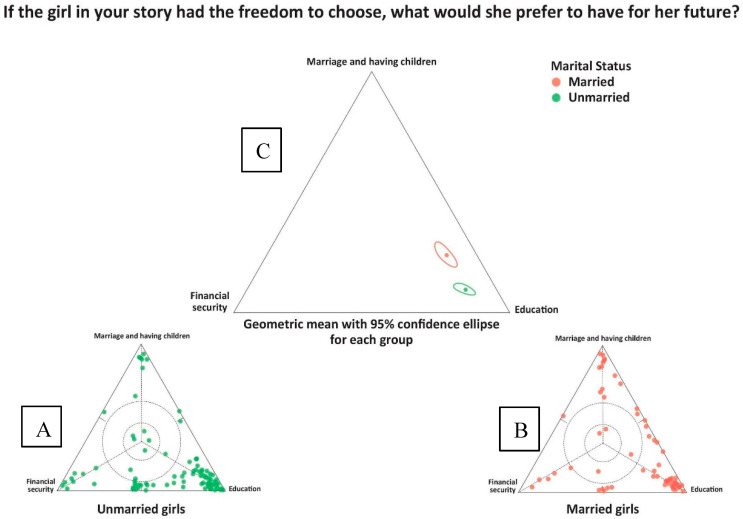
Individual data points: (**A**) unmarried girls, *n* = 111; (**B**) married girls, *n* = 77; and the (**C**) geometric mean with 95% confidence ellipses for all responses within the two subgroups. The question asked was “If the girl in your story had the freedom to choose, what would she prefer to have for her future?”.

The importance of education was laced throughout a large proportion of participants’ narratives and it was closely linked with marital status and with emotional well-being. As such, it is our most prominent theme. As illustrated in Figure 2, girls were asked what they would choose if they could choose among education, financial security and marriage/having children as options for their future. A majority of both unmarried and married girls highlighted education.

While both married and unmarried girls responded towards the education option, girls who were already married were statistically more likely to respond that they may also choose marriage and having children for their future than the unmarried girls whose geometric mean was much closer to the singular “education” vertex. This difference is understandable given that married girls may already have more limited choices with respect to education and may not feel that they will have choices in the future to change that. Qualitative analysis provided more insights into the negative and positive emotions related to education and how these differed between married and unmarried girls.

Frustration was mentioned by 97 of the 188 girls overall (52%) and was the most common negative emotion expressed. Many participants described challenges causing frustration with Lebanese schools including having a different curriculum, certification problems and language barriers, as well as different educational styles and topics compared to Syria. Pressure to leave school in order to work and financially support their families sometimes dampened the girls’ enthusiasm, and frustration about school was also coupled with sadness and anger. A majority of the respondents expressed a desire to continue their education in Lebanon although this was often impossible due to varied social, economic and logistical hindrances. Unmarried girls tended to associate frustration about school with daily challenges they faced when attending school. Some unmarried girls had to drop out of school due to logistical barriers and commented that education was no longer within reach:

*(NarrID 596) “I don’t go to school here, and it frustrates me. I was a good student. Now, I’m forgetting everything, all the material, I wish I could go back to school and start learning again.”*

Married girls, on the other hand, commonly perceived marriage as an alternative to the frustrations faced while attending school. One 17-year-old married Syrian girl in Beqaa (NarrID213) talked about her frustration and her decision to marry as a response:

*“… due to the policies in Lebanon, we are not given any equivalence to our degree. I was in the 9th grade for 3 consecutive years, and I passed every year. But due to the policies set, I couldn’t move up to the 10th grade. I was frustrated from studying the same class for 3 years. Therefore, I got married once I had the chance to.”*

Feelings of shame, fear, humiliation and loneliness were consistently related to experiences of discrimination. For unmarried girls, this was often at school, with experiences of bullying, harassment, mockery and humiliation. Girls expressed feelings of worthlessness and powerlessness. Participants also described hatred directed towards them by educators and Lebanese peers, reporting that they were treated without value and “like an animal”. Other girls described being stigmatized and excluded by Lebanese schoolmates. Humiliation was commonly reported especially by unmarried Syrian girls who were humiliated in school by teachers and peers:

*(NarrID146): “Some people, as I am leaving school, start throwing objects at us and disturb us with their speech. As we enter the school, we go to change into our uniform, but the teacher, as well as the school director, starts shouting at us for being late. In Syria, if we were a bit late to class, the teachers would not say anything, and if they did, they would ask why we were late. But no one ever humiliated us in school the way they do in Lebanon.”*

Many of the Syrian girls in this study experienced fear (*n* = 40) while at school, almost always referencing their peers and teachers as the main source of fear. In some cases, fear was related to experiences of harassment when commuting to and from school, as another unmarried Syrian girl reported:

*(NarrID219): “One evening, me and a group of students were leaving school and had to wait for the bus outside the school campus… During the walk, a car with black tinted windows started following us; every now and then it would shine the front lights and honk. When we reached our camp, the car drove off. My friends and I were terrified. The next day, my mom went and told the Principal what happened and she asked that such incidents never occur again. The Principal apologized on behalf of the bus driver claiming that the driver had a situation and could not come. Of course, we did not believe the Principal. I advise every girl in school to carry a cell phone with her so that if she were ever subjected to such a situation, then she has a means to communicate with her parents and tell them her location.”*

Despite these significant challenges and negative experiences at school, some participants expressed positive emotions with regards to their education. For instance, some girls expressed hope (*n* = 58), sharing aspirations about their future careers, with unmarried girls especially expressing hope to continue their education:

*(NarrID558): “We are not comfortable at all. Our futures have been destroyed and they remain a mystery. I hope that an opportunity will come where I can properly study because I love education and I want to become someone important in society.”*

Feelings of empowerment (*n* = 7) and gratitude (*n* = 21) were most often attributed to being enrolled in formal or informal education, having the opportunity to learn and make friends, and having decision-making power around continuing their education. These emotions were further heightened for girls who had access to good quality education, which was predominantly presented as having “good teachers” who were not abusive or, on rare occasions, were patient with Syrian students, supporting them to adapt to the new educational system, curriculum and language of instruction. Some unmarried girls reported positive feelings about continuing education and having peer support at school:

*(NarrID310): “I am a fourteen-year old girl who is originally from Syria. We left Syria to come to Lebanon at the age of 11, and two months later, we found a school in the hosting country that receives support from the UN, so I enrolled in it. I loved the school, everyone treated us in a good way, I learned French- which I like better than English-, and I made new amicable friends.”*

Married girls related feelings of empowerment and gratitude to having control over decision making about dropping out of school and over whom to marry. In some cases, the husbands supported the needs and decisions of the girls and gave them more freedom than they may have had previously with their parents:

*(NarrID740): “In Syria, I was going to school, and I had available health care services. But when I arrived to Lebanon, I lost a brother, and my parents became sick. Then, I left school, and got married. I was disappointed from more than one person. For a while, I was frustrated, and I felt that everything changed. Now, I am happy. My husband is a nice person, and he gives me all the freedom I need. He didn’t prohibit me from school. But I felt sad for all the people I had lost. I was sad also because my parents are sick. Of course, the education here for Syrians is very weak, and I might not be able to continue my higher education because of that. But at least I am enrolled in school now.”*

### 3.2. Theme 2: Safety Concerns

**Figure 3:**
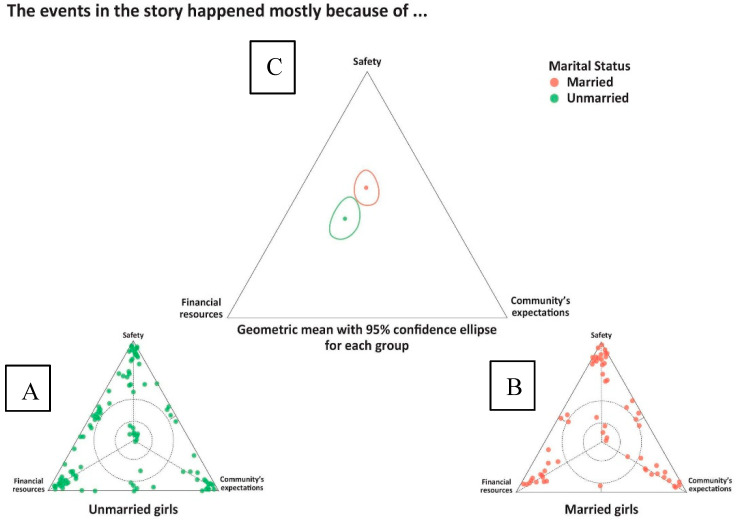
Participant responses for “reasons the events in the story happened”: (**A**) unmarried girls, *n* = 111; (**B**) married girls, *n* = 77; and (**C**) the geometric mean and 95% confidence ellipses for all of the responses in the two subgroups.

As shown in Figure 3, girls were asked whether the events in the shared story happened mostly as a result of considerations for safety, financial resources or expectations of the community.

As illustrated, many of the events shared were perceived at least in part to have been driven by safety considerations, for both married and unmarried girls. Fear for safety was expressed by both married and unmarried girls around incidents such as when men were stopping cars to offer girls rides, men insisting on getting girls’ contact information, and at times forcing girls to get into an unknown car. Unmarried girls expressed fear more often around sexual harassment on the streets and restricted opportunities of leaving their home:

*(NarrI658): “If I want to go to the street, all the guys and men will be saying words to me, and looking at me although I’m wearing decent clothes. There is no safety; the Lebanese guy looks at a Syrian with disrespect, and looks at a girl as if she is nothing, and he gets close to her and tries to harass her, and says disrespectful words. At this point, the girl prefers remaining at home. There are a lot of girls who are 13 years old or so, their parent get them married because they are unable to provide them with all their rights, and to protect them from guys’ harassment, and the education problem, and a lot of other problems.”*

Some married girls expressed these fears as well, especially when being on the streets without their husbands. For married girls, however, there were times when they did not feel safe within their home describing fear in relation to experiences of domestic violence:

*(NarrID275): “I loved a man for a year while we were in Syria. When we displaced to Lebanon, we got married. At first, we were happily married. We had a child, and we were still happy. My sister is married to my brother-in-law. She had a fight with my mother-in-law. So, my husband and my brother-in-law started to beat me. I started to yell, so our neighbor came to help me. But they kept on beating me. My mom picked me up and took me to the doctor. We got a report, and we hired a lawyer to get my son back. Now, my son is with me, and I returned to my parent’s house waiting to get divorced.”*

Safety concerns and parents forcing unmarried girls, especially, to stay at home was also causing frustration (*n* = 97) and loneliness (*n* = 68), as well as feelings of unequal gender-based rights. Some girls felt alone in Lebanon if part of the family was still in Syria. Both married and unmarried girls described feeling isolated at home and frustrated when not able to go out unless accompanied by an adult male family member. Unmarried girls were at times coerced by their parents to stay at home, which meant that for a majority of the girls, they were not able to leave the camps:

*(NarrID696): “… When we came here to Lebanon we started living in a tent, in a camp, away from everything. We can’t just leave and roam around because people talk and it bothers us. We don’t have school… there is no country like Syria, we don’t have diplomas. I can’t go to the market and go on walks with my friends because my parents get worried. I can’t even get out of the camp. My life is limited to this camp, this tent and that’s it.”*

These negative feelings of unmarried girls were also associated with a strong dependency on their parents, who, in some cases, overregulated their daily lives without addressing the needs of the girls. Married girls hoped to be less ignored and treated well by their husbands. However, they also reported dependency and restrictions in movement within their marriage:

*(NarrID441): “I was pressured at my parent’s place. I didn’t feel any warm-heartedness from my parents and siblings. When a girl is pressured at her parent’s house, she would choose to get married no matter whom the husband is. My parents pressured me a lot, and they watched my every step. They interfered in everything, and they tried to control me in every way.”*

*(NarrID537): “My husband prohibits me from going to school and from going out. I cannot go out without my mother. We go out three days per week as a couple.”*

Safety concerns in some cases led to coerced marriage or girls intentionally deciding to marry early. These marriages were often described as unsatisfying and unfulfilling, with experiences of physical, verbal and/or emotional abuse. As a result, married girls sometimes felt overwhelmed, bothered and/or uncomfortable due to a lack of independence and not able to decide about their own future. Some of these narratives concluded with a feeling of helplessness expressed by the women and girls, whereby they felt that they were unable to leave the unhealthy and/or violent relationship primarily due to fear of social pressure and negative repercussions if they decided to leave:

*(NarrID938): “We came here to Lebanon and we lost hope, trust and love... I got engaged but not because I love him but just because I’m at an age where I should find the partner of my life. I can’t seem to be happy or comfortable with him, there’s no harmony. But, I only imagine the consequences if I leave him, society will judge me, it won’t be accepted, I don’t know if we’ll ever live in harmony. I can’t leave him now.”*

Despite a lack of safety being faced after displacement into Lebanon, some stories contained gratitude (*n* = 21) including feeling safe from the war in Syria:

*(NarrID1319): “In Syria, we weren’t comfortable due to the war. Here, our conditions aren’t good but at least we are safe, and there is no war. More than one family is living together in a small house. My husband and I live with his parents’ since we cannot afford to pay rent. They are approving of the girls’ marriages at an early age to secure their futures.”*

Other stories highlighted how the girls were grateful and happy (*n* = 28) in their current lives, even among married girls despite being married as child brides. In the majority of these cases, the feeling of happiness was linked to marrying someone they love and safety as well as financial stability within marriage, as well as having a say in whom and/or when to marry:

*(NarrID444): “In Syria, I was enrolled in grade 10 at school. When we displaced to Lebanon, I came to work. Several men have asked to marry me, but I would refuse. Until, I accepted to marry my current husband, and I am very happy. We have a child, and I am pregnant.”*

Moreover, some girls reported feelings of empowerment (*n* = 7), where in one case, a 15-year-old married girl was able to take action to leave a violent marriage:

*(NarrID271): “I didn’t get engaged, and I didn’t do a wedding. We were married in four days only. Problems started immediately. I lived with him for 20 days only. He used to beat me, he wanted to send me to turkey, he wanted me to work as a dancer, and he wanted to take me away from my parents. One day, at 11 pm, I ran away from him. People I know helped me to get from where he lived to my parent’s village. Then, my parents helped me to get a divorce.”*

### 3.3. Theme 3: Peer Support

The third overarching theme that emerged from the data was the importance of peer support for girls’ emotional well-being. Some participants had lost close family members and separated from friends during or after the war, which affected their emotional well-being. Expressions of sadness (*n* = 58) and loneliness (*n* = 68) were often linked to experiences of exclusion as being a displaced person in the host country, and the restricted lives that adolescent girls are often forced to lead. However, it is noteworthy that narratives regarding peer support are different for married and unmarried girls. Married girls talked about separation from friends and family and separation from the lives that they knew in Syria, as the turning point, and an impetus to get married. Unmarried girls’ stories shed light on the importance of having peers and a support system:

*(NarrID696): “I was living happily in Syria, I used to go with my friends to school peacefully not afraid of anything, we used to go to the beach and the gardens and we were happy. We used to go out with my grandparents, my parents and my friends. When we came here to Lebanon we started living in a tent, in a camp, away from everything... I can’t go to the market and go on walks with my friends because my parents get worried.”*

This kind of support allows them to take control of their lives, and thus feel empowered:

*(NarrID522): “I came to Lebanon when I was 11. I have made new friends, and now I have every Thursday to look forward to when I go out and see them.”*

Both married and unmarried girls often expressed gratitude having escaped the war in Syria, and gratitude for being together and alive with family and friends, despite the challenges faced as a refugee in Lebanon. This 17-year-old unmarried Syrian girl arrived in Lebanon at 11 years old:

*(NarrID522): “Everything in my life changed. I love to cook, and hope to learn it professionally. I have made new friends, and now I have every Thursday to look forward to when I go out and see them. Otherwise, I am always at home. I love that I am healthy and I wish that my situation gets better.”*

### 3.4. Theme 4: Longing for Life Back in Syria

In relation to this fourth important but slightly less prominent theme, the emotions of married and unmarried girls were similar. There is a general shared longing to return to their life before the war. Many participants compared their living situations in Syria and Lebanon and felt that life in Syria was easier and more desirable before the war. Both married and unmarried girls voiced immense sadness (*n* = 58) about adapting to a new life in Lebanon and expressed great hope to return to Syria:

*Unmarried (NarrID1549): “I wish to return to Syria and that life in Syria returns to what it was before, I loved it more than life in Lebanon. I love to learn and get an education and so do my parents, and they wish that I become a successful working woman someday, and I hope I can grant them that wish.”*

*Married (NarrID688): “We noticed how life changed drastically, in Syria I was living happily and peacefully… I hope that we could someday go back to Syria, so that we could live our lives, that every Syrian girl could live her life without suffering, I suffered a lot.”*

Feelings of despair also resulted from experiences of exploitation at work since economic hardship in Lebanon has led many formal refugees or informal migrants to agree to difficult working conditions that are causing psychological stress:

*Unmarried (NarrID1501): “Since I am Syrian, they want to give me more work than I can handle. They want to torture the Syrians that are arriving to Lebanon; this is how they think. That’s basically why I was going through psychological pressure. I used to arrive home crying. Due to this pressure, I was getting ill a lot, and I had an accident while I was working at home.”*

Despair has also resulted from incidents of discrimination and xenophobia for both married and unmarried girls. Respondents note that they are foreigners in a foreign land. Maltreatment by other members of the community was commonly mentioned. Participants shared feelings of frustration associated with a lack of official registrations or difficulties in renewing travel documents and/or crossing the Syrian/Lebanese border as a result of changing policies:

*Married (NarrID430): “One time, I had to go to the hospital for my son. On my way there, a car started driving slowly behind me, and inside was a man who was asking me if I wanted to get in the car with him so he can drop me off. I ignored him, but he continued to bother me, so I went back home to get my husband to go with me. I couldn’t find my husband so I took my brother-in-law. Since that incident, I have not dared leaving the house without my husband or brother-in-law. More so, I did not tell my husband about what happened because he has a temper and will make a problem out of this, and since we are Syrians, we will be blamed and accused.”*

Girls are bothered by poor living conditions and feel humiliation for being a refugee. Some have lost hope for a better life outside the tent camps:

*(NarrID141): “We were very hopeful of becoming recognized members in the community, but now, we do not have that hope anymore… During the holidays, we wished to be like all the Lebanese women who were able to come and go as they wish. However, we did not go anywhere, we stayed inside the house, as we have done for the past three or more years …We do not leave the house because our parents keep telling us that there is no security for us since we are living on other people’s land. When we are walking on the streets, we do not feel like we are walking in our countries. The looks we receive make us prefer living amid the war in Syria. Their looks humiliate us, and so does the word “Syrians”.”*

Despite the longing that many girls expressed for their life in Syria, it is worth highlighting that in few cases, the girls expressed gratitude (*n* = 21) as a form of acceptance of their current realities and thanking God for what they have, despite being explicit regarding their current suffering. In addition, the narratives of some girls focused on hope for more acceptance and respect, and the power to fight against discrimination and stigmatization in the host country. Moreover, many girls expressed the wish that the Syrian civil war and humiliation would end, so that they could return back home to their old happy and stable lives:

*(NarrID688): “Here I only spend time at home, I don’t go out, I don’t have friends... I’m not informed about what’s been going on outside, I thank God though, we are still living a somewhat better life regardless of some difficulties. I hope every girl gets to live her life, a life of her choice.”*

## 4. Discussion

### 4.1. Summary of Findings

This study explored emotional well-being among female Syrian adolescents displaced to Lebanon. Using SenseMaker, a total of 188 first-person stories and their accompanying quantitative survey data were captured. Overall, both unmarried and married girls were more likely to associate their stories with negative emotions than positive ones. The qualitative thematic analysis indicated that negative feelings such as frustration, anger, and loneliness were most prominent. It is notable, however, that for both married and unmarried girls, despite often challenging living situations and various hardships, a subset of the narratives was hopeful and expressed happy feelings.

Four overarching themes emerged as being central to positive or negative emotional well-being for Syrian girls in Lebanon: access to education, concerns about safety, peer support, and longing for life back in Syria.

Upon closer inspection, there were some differences in emotional well-being and its determinants between married and unmarried girls, although these were not as distinct as one might predict. Barriers to accessing education were linked to frustration for both married and unmarried girls. However, because unmarried girls are more likely to be in school or attempting to continue their education, the source of their frustration often differed from that of their married peers. Unmarried girls more frequently mentioned negative experiences of public maltreatment and bullying, xenophobia and humiliation by teachers and peers in schools which is in line with other research findings [7,12,44]. Some unmarried girls had positive educational experiences and expressed feelings of gratitude and empowerment, as well as feelings of inclusion and receiving support from their peers. These findings correspond with findings from Chahine et al. (2014) [12], who describe the relationship between the Syrian refugee youth and the hosting community as ambivalent, yet two-thirds of Syrian adolescents made friendships with Lebanese students in school compared to 30% among out-of-school youth. Nonetheless, these girls also faced many challenges including logistical issues, adapting to a different curriculum and psychosocial pressures in the form of discrimination and humiliation by teachers and peers [7,12,41].

Married girls, in contrast, related their frustration to restricted opportunity to continue school after marriage. It is understood that discontinuing education can result in devastating longer-term outcomes among Syrian girls [9,10,12,15]. Yet, it was within this theme that married girls seemed to have greater hope and stronger emotional well-being. For instance, some married girls expressed positive feelings about their marital relationships and the increased sense of safety that marriage had brought them. In all of these narratives, the positive emotions were linked with having a supportive and understanding partner and some married girls associated feelings of happiness with having a husband who could protect them. However, these results are based on marriages which are perceived by girls as an independent decision and as a better alternative to the current situation. Several studies have found that child marriage and limitation of girls’ mobility are common coping strategies families employ when dealing with poverty and perceived, or real, safety concerns in situations of displacement [7,15,27,29]. When acknowledging that child marriage is a violation of human rights and of the rights of the child, it is vital to also understand that in light of the dire conditions in Lebanon, specifically in relation to the inability to access quality education, marriage was perceived by some of the girls, and their families, as a better alternative [15]. This finding supports the necessity for a nuanced understanding of child marriage, particularly in settings where girls’ experiences of marrying early are embedded within an overwhelming hardship of having fled war and of facing difficulties in the hosting country such as poverty, vulnerable housing, insecurity, and a lack of access to education [11,15,17,33,38]. An intersectional critical feminist analysis permits this nuanced understanding of early marriage among displaced women and girls, in comparison to historical representations of victims. It challenges a universal “refugee women’s experience”, instead offering concepts of resilience and resistance in the face of discriminatory practices or victimizing narratives surrounding their lives in the camps and urban settings. A critical feminist approach provides the tools to identify the global power structures and local vernaculars of ethnicity, language, religion, and socio-economic status that inform the circumstances of being a refugee woman [57]. With regard to Syria, not only is there a distinct culture, lifestyle and set of customs, but the ongoing war—as well as the displacement and resettlement that ensued—has challenged these, resulting in an alarming increase in mental health problems among adolescents [33,38,58,59].

Displaced adolescent girls often lose their peer groups and may experience feelings of loneliness [12]. This research confirms others [17] urging that the girls’ own understanding of their situations must be heard, and that peer relationships are essential, particularly at this stage in development [15,17].

Concerns about safety since displacement to Lebanon were important for all girls in this study. Living in informal tented settlements and overcrowded or substandard housing had exacerbated stress [14,39,40,42]. Both married and unmarried girls reported restricted movement, which was a source of frustration and loneliness, and a general lack of independence [12,15]. Unmarried girls were controlled by their parents, while some married girls discussed having their movement restricted by their husbands. Both groups acknowledged a lack of social support and felt a need to connect with peers, as well as a general feeling of isolation and sadness. Our findings around peer support add to existing research on the need for friendships with both migrants and locals for Syrian girls in the hosting country [12,17].

Both married and unmarried girls expressed a desire to return to Syria and the lives that they had left behind. No significant differences were present between the two groups in terms of types of expressed emotions in this regard, and both often referenced negative emotions. Many girls shared an immense sadness about having to adapt to a new life in Lebanon and expressed great hope to return to Syria. Sadness was often associated with missing certain aspects of their lifestyles before forced displacement, while frustration was often referenced around the experience of xenophobia. Nonetheless, girls equally expressed gratitude for having escaped war in their country despite the poor living conditions in Lebanon [60,61].

### 4.2. Strengths and Limitations of the Study

This study has some noteworthy strengths that reside largely in its methodology. Firstly, it is based on findings from both qualitative and quantitative data providing both breadth and depth to our understanding of adolescent girls’ emotional well-being during displacement. Secondly, the study is unique in that it was able to collect a significant amount of data from a hard-to-reach population. We were able to hear directly from girls themselves, empowering them to voice their experiences and facilitating agency to meet participation rights. Lastly, the study was able to capture first-person narratives from both married and unmarried girls so that emotional well-being and its determinants could be explored by marital status.

Despite its strengths, the study also presents a few limitations that should be taken into account when interpreting the results. The data were collected through convenience sampling, which has the possibility to create a participation bias and may negatively affect the generalizability of findings. Nonetheless, given the population and the exploratory nature of this study, this type of sampling was deemed appropriate in such circumstances and provides a basis on which future research can be developed. Furthermore, despite using a ‘participant-friendly’ tool [16], the qualitative data used in this study consisted of the collection of short narratives without probing questions or full interviews. Thus, the depth of understanding gleaned from the qualitative data is somewhat limited. In addition, participant narratives were transcribed from Arabic into English, and then analysed. It is important to consider that there may be language- or culturally-specific nuances that may have been missed, despite having taken lengths to minimise any impact to the interpretation of findings. Unfortunately, we did not collect demographic information about the age differences between married Syrian adolescent girls and their husbands, and therefore we were not able to explore this aspect in our analyses. Finally, the quantitative questions were predetermined and closed ended—both of which could limit the emergence of new insights or themes. The quantitative results can only be interpreted as possible trends and are not sufficient for generalisation.

### 4.3. Implications of the Study for Practice and Research

Our findings highlight the importance of designing more specific interventions to address the needs of both unmarried and married girls. Since unmarried girls discussed isolation, discrimination, and a loss of friendships, they might benefit from a greater exchange between migrant and local adolescents, supporting their need for belonging with peers and through organized activities. Of paramount importance is providing continuous, freely accessible, age-appropriate education for girls to address a plethora of their concerns. Collaborations between NGO centers and educational institutions might facilitate opportunities for Syrian girls attending school to engage with Lebanese adolescents (i.e., artistic or sports projects, tutoring, and engaging in public work). Educational opportunities would offer girls safe spaces, improve future prospects for employment, facilitate social contacts, and provide a sense of purpose. The provision of schools and educational spaces should consider the barriers that prevent girls from attending school, such as geographical location and fear of harassment. Interventions that address child marriage in humanitarian settings must also acknowledge girls’ loneliness, safety concerns and their experiences of loss, and include social networking and engagement opportunities.

Moreover, both married and unmarried Syrian adolescent girls in Lebanon are likely exposed to diverse forms of GBV (e.g., domestic violence, harassment, and exploitation). Violence prevention programs should target boys and girls in schools and community groups as well as awareness raising in other community spaces. A shift in gender roles and expectations is important across refugee and non-refugee populations to enable both sexes to be more independent and to have opportunities and agency that are not undermined by fixed expectations of society based on gender [30,32,33]. Moreover, integrative educational programs should also devise inclusive approaches to involve the students’ parents and target this group in awareness- and skills-building interventions such as parenting skills training. This would complement girls empowerment curricula [62]. Prevention programs designed to address GBV must challenge the socialization of men towards the use of violence and raise awareness about the negative effects of GBV. For example, in earlier work, non-violent practices and discourses by Syrian men were defined by their ability to foster peace and shun violence while affirming that violence was not an inherent attribute of manhood [63]. There are promising programs which aim to alter social norms and empower bystanders to intervene to stop GBV [64,65]. Cash-based education programming like the No Lost Generation (NLG) initiative are promising and their development and testing further supported. The NLG program covers the cost of commuting to school while compensating households for lost income if their children do not work. This intervention has been shown to increase school enrolment across all of Lebanon’s governorates [66].

The findings also highlight the vital need for adolescents, especially girls, to access mental health and psychosocial support (MHPSS) to alleviate the negative emotional impacts of displacement. One study in Lebanon measured Syrian refugee children’s access to health services and averaged it at 4.16 visits per child per year, in line with the SPHERE standards during humanitarian emergencies [67]. However, these numbers were significantly lower when compared to the accessibility of host children to health services, whereby only 11% of Lebanese children did not access services as compared to 25% of refugee children [67]. Despite the lack of Lebanon-specific data exploring the needs of refugee children in terms of MHPSS services, a systematic review emphasized the need to provide assistance and suggested designing simple interventions to increase children’s resilience [68].

One such intervention, previously used and tested among Palestinian refugee children in Lebanon, could be replicated. It is called ‘Qaderoon’ (We Are Capable) and it targets children (11-14) with an aim of improving mental health of refugee children while increasing their commitment to school [69]. As part of WHO efforts to mainstream low-intensity global programs within humanitarian contexts, a few of its programs have been rolled out including Program Management plus (PM+), Self Help plus (SH+), and Early Adolescent Skills for Emotions (EASE) [70,71]. Only EASE is tailored to the needs of the adolescent target population of this study. It includes four sessions delivered by non-specialized personnel [71]. In 2019, Brown et al. (2019) announced the first randomized control trial (RCT) aimed at assessing the effectiveness of the program in Lebanon and Jordan, and 2020 findings demonstrate that the adaptation process in Lebanon was feasible and received high acceptability by youth and their caregivers [72]. Two other interventions, Therapy by Repeating Phrases of Positive Thoughts (TRPPT) and Cognitive and Positive Psychotherapy (CPPT), evaluated the effects of specialized therapeutic methods and found positive outcomes. These methods have been shown to reduce war-related mental health challenges and have achieved positive acceptance among study participants [73]. It is worth noting, however, that findings of a review of MHPSS services for Syria communities highlighted that from the refugee populations’ perspective, urgent therapeutic needs were often as simple as having a space to share past hardships as a means to regain control of their current reality [74]. As such, attending to these unique needs often does not require trained or specialized clinical mental health staff (already in shortage), but may work best through non-specialized peer-led or community psychosocial support interventions that can enhance individual abilities and coping skills while simultaneously working to rebuild social bridges and support networks [74]. Further research is needed to assess the effectiveness of interventions that are contextualized for the age group, that ensure cultural relevance, and that establish good relations with parents to ensure youth’s continued commitment [75]. That being said, findings from this study can specifically guide the development of gender-sensitive approaches that are also inclusive of married girls in addition to unmarried girls in a displaced environment.

In establishing policies with an intersectional feminist approach, consideration of access to power would be pivotal, with acknowledgement of migrant status, culture and traditions as they intersect with gender [32,33]. Policies would promote more knowledge about the individual needs and vulnerabilities of migrant, adolescent girls compared to others in Lebanese society. Feelings of “happiness” for being saved, or of “poverty” reveal more about the conditions of their new lives, and not the nuance of present vulnerability or the possible future for refugee and migrant women and girls. Precarious livelihoods and a semi-legitimate socio-legal status are increasing the risk for severe labor exploitation and also lead to the emergence of human trafficking of young girls and women for the sex trade [76]. Displaced Syrian adolescent girls and women are exposed to language issues in Lebanon due to their poor proficiency in English or French [77,78] and thus often face challenges in school or work in poorly paid informal sectors such as housekeeping or childcare duties, which further increases their economic dependency, and precarity has to be considered [35,79,80]. The lives of displaced refugee communities in the Middle East are characterized by poverty and insecurity [7,15,38] and yet there is a gap of knowledge not only about gendered disadvantages such as exploitation in marginal sectors or through clandestine marriages and forced sex trade but also gendered advantages such as empowerment in a potentially more liberal society. Research must focus on the differential impact of war and displacement on women and girls, of different ages, in order to uncover gender-based power relations and deconstruct the so-called gender-neutral approach to precarity in forced migration contexts.

Given the context of this study, there is a great need for future investigation. With no resolution in sight for Syrian refugee and migrant families in Lebanon, and the ever-changing socio-economic landscape, using research as a vehicle for (open-ended) investigation of girls’ concerns is invaluable. The results of this study can act as a basis for further exploration.

## 5. Conclusions

Displacement appears to be linked to the emotional well-being of both married and unmarried Syrian girls in Lebanon. Programming to address the health and development needs of these adolescent Syrians has been challenging partly because of the dispersion of the migrant population due to the no camp strategy by the Lebanese government [5,6]. As such, understanding the unique experiences of adolescent girls, specifically after displacement, is vital when designing relevant and effective interventions to meet their needs. Moreover, comprehending the complex emotions associated with these experiences not only humanizes the humanitarian response but also provides an additional layer of understanding. Consideration of marital status and its intimate relationship with security, education and emotional well-being of refugee girls in Lebanon is essential.

An increasing lack of educational opportunities for adolescents, feeling unsafe, feeling isolated from peers and longing to go home all contribute to negative emotional well-being. While the differences in emotional well-being and response between married and unmarried girls were not great, there were some important nuances in understanding about the sources of emotional support or strain that were brought forward by this study. These differences point to the need for targeted and well-informed interventions.

Despite the wealth of challenges faced by both unmarried and married adolescent girls, a range of positive emotions were also expressed including feelings of gratitude and hope for a better future. We join with these young people in hoping that the situation will improve and that further support for their emotional well-being will be realized.

## Figures and Tables

**Table 1:** Characteristics of the Study Sample of Syrian Adolescent Girls.

Characteristics	Syrian Adolescent Girls n (col %)	Unmarried Syrian Adolescent Girls n (col %)	Married Syrian Adolescent Girls n (col %)
**Age**			
13–17	188	111 (59%)	77 (41%)
**Location in Lebanon**			
Beqaa	93 (49%)	52 (47%)	41 (53%)
Greater Beirut area	39 (21%)	32 (29%)	7 (9%)
Tripoli	56 (30%)	27 (24%)	29 (38%)
**Time in Lebanon (years)**			
less than 1 year	16 (9%)	8 (7%)	8 (10%)
1–3	61 (32%)	36 (32%)	25 (32%)
3–5	111 (59%)	67 (54%)	44 (57%)

Each cell indicates the number of shared stories in that category and the column proportion.
